# Effects of a Text Message-Based Lifestyle Intervention on HbA1c and Health Behaviors in Older Adults with Prediabetes

**DOI:** 10.3390/nu18040682

**Published:** 2026-02-20

**Authors:** Jung Hun Lee, Hee Jin Kim, Kang Hyun Lee

**Affiliations:** 1 Department of Digital Healthcare Convergence, Songho University, Hoengseong 25242, Republic of Korea; jh.lee@songho.ac.kr; 2 Department of Emergency Medicine, Yonsei University Wonju College of Medicine, Wonju 26426, Republic of Korea; hj.kim@yonsei.ac.kr

**Keywords:** mHealth, prediabetes, digital health intervention, text messaging, self-management

## Abstract

Background/Objectives: Prediabetes (PD) does not always progress to type 2 diabetes; however, lifestyle modification and weight loss are recommended to prevent disease progression. This study aimed to determine whether a text message-based intervention for older adults with PD in South Korea could reduce glycated hemoglobin (HbA1c) levels and promote healthy habits. Methods: A randomized controlled trial with two parallel groups was conducted and approved by the Institutional Review Board of Wonju Severance Christian Hospital (IRB No: CR321379). A text message intervention was provided only to the intervention group for six months, while the control group was advised to maintain their usual daily routines. The text messages covered the following categories: nutrition, exercise, medical knowledge, lifestyle, and self-reflective thoughts. Results: A total of 96 participants aged 50 years and older underwent clinical measurements, completed the Korean Health Habits Questionnaire, and were assessed for quality of life at baseline and after the intervention. HbA1c, waist circumference, and fasting blood glucose decreased significantly in both groups (*p* < 0.05). The intervention group demonstrated significant improvements in body mass index, low-density lipoprotein cholesterol, total cholesterol, and milk consumption. In contrast, instant food consumption increased significantly in the control group, resulting in significant between-group differences (*p* < 0.05). In addition, the frequency of late-night eating increased significantly within the control group (*p* < 0.05). Conclusions: Text message-based interventions may serve as an effective adjunctive self-management strategy to improve HbA1c levels and promote healthy habits in older adults with prediabetes.

## 1. Introduction

Prediabetes (PD) is a metabolic condition in which blood glucose levels are above the normal range but below the threshold for diabetes [1]. In 2024, an estimated 487.7 million adults, corresponding to 9.2% of the global adult population, were reported to have impaired fasting glucose (IFG), a major component of PD [2]. The prevalence of PD has been rising worldwide. For example, the U.S. Centers for Disease Control and Prevention reports that about 98 million American adults have prediabetes, which corresponds to more than one in three adults in the United States [3]. In South Korea, the high prevalence of PD is also a major public health concern. According to the Diabetes Fact Sheet in Korea 2024, the prevalence of PD among adults aged 30 years and older has surged to 41.1%, with the rate exceeding 47.7% among those aged 65 and older [4]. It suggests that older adults may constitute a particularly high-risk population for PD, potentially associated with age-related metabolic changes and the rapid aging of the population [5]. Additionally, approximately 5–10% of people worldwide with PD progress to diabetes annually, and approximately 37% and 70% of individuals may be at high risk of developing type 2 diabetes mellitus (T2DM) or related complications within four and ten years, respectively [6,7]. Among adults aged 65 years and older worldwide, the prevalence of IFG is 29.6%, and this burden continues to increase. Considering the potential reversibility of IFG, in contrast to the largely irreversible nature of diabetes, PD has received more widespread attention [6].

The American Diabetes Association (ADA) 2025 guidelines emphasized the importance of preventing diabetes and stated that patients with PD should be regularly monitored for type 2 diabetes at least once a year [8]. Patients with PD are a high-risk group for diabetes, and it is recommended that all patients with PD implement diabetes prevention strategies. Many studies have used glycated hemoglobin (HbA1c) as a clinical indicator to assess glycemic status, and an HbA1c level of 5.7% to 6.4% is considered a diagnostic criterion for PD [2,8]. Compared with fasting plasma glucose (FPG), HbA1c reflects average plasma glucose levels over the preceding 3–4 months [9]. Additionally, the HbA1c test is convenient and is not influenced by short-term external factors, such as recent meals or environmental conditions, and is therefore considered a reliable measure [10].

Several studies have reported that PD leads to an increased risk of macrovascular disease, chronic kidney disease, diabetic retinopathy, and other diabetes-related complications [11,12,13]. However, as PD does not always progress to T2DM, it is recommended to prevent disease progression through lifestyle modification, including dietary changes, increased physical activity, and weight reduction [14,15]. In particular, maintaining healthy lifestyle behaviors may be difficult for older adults due to physical limitations, social isolation, and reduced access to structured health promotion programs [5,16]. Lifestyle behaviors are also influenced by regional factors. People living in cities and suburbs tend to refer to nutrition labels more often than rural residents. Regional differences show associations among dietary factors, behavioral differences, and chronic diseases such as diabetes [17]. By modifying the diet and forming a healthy lifestyle, including regular physical activity, regional health disparities can be reduced.

Text-message-based lifestyle interventions serve as a cost-effective and widely accessible strategy and have demonstrated potential in the prevention and management of diabetes with relatively low resource requirements [18]. In addition, because text messaging requires minimal technological literacy and is compatible with basic mobile phones, it may be particularly suitable for older adults [19]. Previous studies indicate that such interventions may support adherence to self-management behaviors, including lifestyle modification and routine health monitoring, and are associated with modest improvements in glycated hemoglobin (HbA1c) levels [20,21]. More recent approaches incorporating two-way or interactive messaging have been reported to enhance participant engagement by allowing individualized feedback and supportive communication, which may facilitate motivation for sustained lifestyle change [22]. By avoiding the technical complexity and financial burden associated with more advanced digital health platforms, text-message-based interventions may offer a practical approach to extending health education and preventive care to underserved and socioeconomically disadvantaged populations [23].

In this study, we aimed to examine whether an intervention using mobile phone text messaging for older adults aged 50 years and older with PD living in rural South Korea could (1) improve HbA1c levels, thereby lowering the risk of diabetes, and (2) support the formation of healthy lifestyle habits.

## 2. Materials and Methods

### 2.1. Study Design

This study is a secondary analysis of data derived from a previously conducted randomized controlled trial. The original clinical trial was conducted at Yonsei University Wonju Severance Christian Hospital (Wonju, Republic of Korea) from December 2019 to June 2020, using a two-parallel-group design and was registered at the Clinical Research Information Service (Registration No.: KCT0005783). The control group received only usual care during the six-month study period, while the intervention group received text messages for diabetes prevention. The protocol was approved by Wonju Severance Christian Hospital (IRB Approval No.: CR321379). All personal identifying information collected in this study was anonymized and coded. Participants were informed of the study purpose and data usage, as well as their right to withdraw consent at any time. Only those participants who provided informed consent were recruited for this study.

### 2.2. Participants

**Figure 1:**
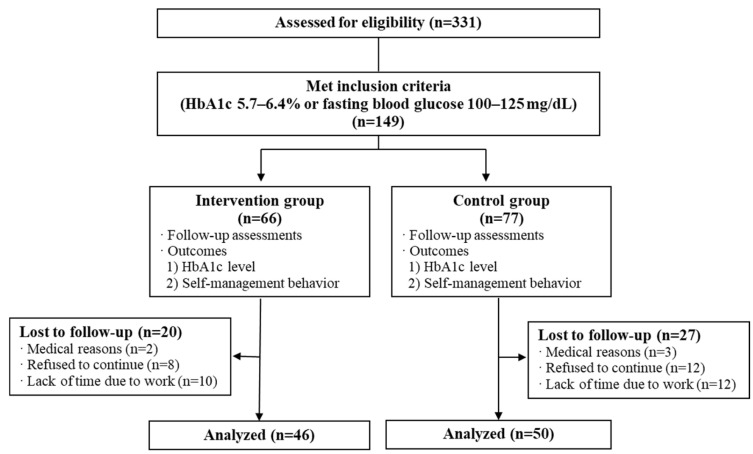
Flow of participants through the study.

The criteria for the selection of study participants were as follows. First, participants with PD were defined as participants with an HbA1c level of 5.7–6.4% or a fasting blood glucose (FBG) level of 100–125 mg/dL, according to the ADA definition. The study participants were as follows: (1) adults aged 40 to 80 years old; (2) those who met the HbA1c criteria for prediabetes; (3) previous participants in the Wonju–Pyeongchang Cohort Project conducted by the Korea Centers for Disease Control and Prevention; (4) participants in this clinical trial who provided informed consent; and (5) participants who understood the education and instructions and could participate in the entire study period (Figure 1).

### 2.3. Intervention

For six months, self-management for diabetes prevention was recommended through mobile phone text messages, and support was provided to motivate and promote the formation of healthy habits. Messages were sent at least three times a week, and five categories were used. They focused on improving lifestyle habits, such as eating healthier foods, getting more active, and reducing unhealthy foods. Text messages were automatically sent between 8 a.m. and 5 p.m. at an individual’s preferred time or at a time determined by the research coordinator, according to the coordinator’s availability. The composition categories were divided into nutrition (11 messages, 23%), physical activity (7 messages, 15%), medical knowledge (19 messages, 40%), lifestyle (6 messages, 12%), and self-reflective thoughts (5 messages, 10%). The subcategories consisted of awareness, alternative choice, change, risk control, support from others, and diabetes knowledge. The individual’s name was automatically applied to each message to increase immersion in the study. Example messages are illustrated in Table 1.

### 2.4. Measurements

#### 2.4.1. Korean Style Dietary Habit Questionnaire

To investigate changes in eating habits, a South Korean–style dietary habit questionnaire reflecting South Korean food culture was used [24]. The South Korean–style dietary questionnaire was developed by the Delphi technique and is a standardized questionnaire that has been verified for South Korean adults. The questionnaire consists of 25 questions regarding health habits (number of usual meals, overeating frequency, and the number of late-night meals) and the frequency and type of food consumption (such as grains, meat, fish, fruit, vegetables, milk, instant foods, and fast food; rating options: “once a day/week” or “never,” etc.).

#### 2.4.2. Measurement of Clinical Values

All study participants had clinical measurements at the first visit and at the six-month follow-up. Blood tests (FBG, HbA1c), blood pressure, weight and height, and waist circumference were measured. For accuracy, blood pressure and heart rate were measured in a sitting position after resting for more than 10 min. Blood samples were analyzed through an external authorized institution. Participants also completed a self-report questionnaire on demographic characteristics, such as age, gender, and monthly income. Monthly income was investigated to verify its established relationship with diabetes [25].

#### 2.4.3. Quality of Life Assessment

For quality of life, the most widely used and validated quality of life assessment questionnaire (EQ-5D-3L) globally was used [11,13]. It is a standard questionnaire designed to measure the patient’s self-reported health status, indicate self-perceived health status, and includes five categories: mobility, self-management, daily life (such as work and study), pain or discomfort, and anxiety or depression [12]. Scores range from 1 to 3, with values closer to 1 indicating better health. Index values range from −0.171 to 1, with values closer to 1 indicating better health [10]. This questionnaire can verify physical exercise ability and changes in mental health, such as anxiety or depression.

#### 2.4.4. Statistical Analyses

All data were collected using a face-to-face questionnaire survey with participants. For the participants, descriptive statistical analyses of general characteristics were performed for continuous data and expressed as means and standard deviations. Paired *t*-tests were used to compare clinical indicators before and after the trial, and independent *t*-tests and chi-squared (χ^2^) tests were performed to compare differences between the groups. For the quality-of-life evaluation, Fisher’s exact test was used in cases with expected cell counts of less than five. Statistical analyses were performed using SPSS version 26.0 (IBM Corp., Armonk, NY, USA), and all statistical significance levels were set at *p* < 0.05.

## 3. Results

### 3.1. Baseline Characteristics of the Participants

Of the 96 participants, 46 (48%) were in the intervention group, and 50 (52%) were in the control group. The average age of the intervention group and the control group was 65.4 ± 5.5 and 66.9 ± 6.5, respectively, and there was no statistically significant difference between the two groups (*p* = 0.247). Regarding clinical values, there were no statistically significant differences between the groups for the variables presented in Table 2. At baseline, no significant differences were observed between the groups in any clinical variables except for triglycerides (Table 3). It was also confirmed that there was no difference in educational level (*p* = 0.724) or average monthly income (*p* = 0.191) between the groups (Table 2). Healthy lifestyles were measured by the frequency of consumption of healthy foods (such as grains, fish, fruits, vegetables, and milk) and unhealthy foods (such as fatty foods, instant foods, and junk food), as well as eating behaviors (including late-night eating, eating out, and overeating), and no significant differences were observed between the groups at baseline (Table 3).

### 3.2. Changes in Health Habits and Clinical Results

To examine the effect of the message intervention, clinical values measured at the first and last visits and measurable changes in healthy lifestyles were analyzed (Table 3). HbA1c, waist circumference, and FBG were significantly decreased in both groups after the clinical trial. Body mass index (BMI), low-density lipoprotein cholesterol (LDL-C), and total cholesterol significantly decreased from baseline to six months in the intervention group, and there was no significant difference between the groups. LDL-C and total cholesterol did not differ significantly between the two groups at baseline; however, statistically significant between-group differences were observed at six months (*p* < 0.05). In the intervention group, male participants showed a statistically significant reduction only in waist circumference, whereas female participants demonstrated significant reductions in waist circumference, LDL-C, total cholesterol, and FBG. Regarding lifestyle behaviors, a notable change was observed in the frequency of instant food consumption. There was no significant difference between the intervention and control groups at baseline, but a significant difference was observed at six months (*p* < 0.05). In the intervention group, milk consumption increased significantly (*p* < 0.05). In the control group, fruit consumption increased; however, the frequency of late-night eating also increased significantly (*p* < 0.05). In the intervention group, no statistically significant changes were observed in male participants. In contrast, female participants showed a decrease in vegetable consumption and a significant increase in milk consumption (*p* < 0.05). In the control group, late-night eating increased significantly in men, whereas women demonstrated statistically significant increases in instant food consumption and take-away food frequency (*p* < 0.05).

### 3.3. Quality of Life Assessment Results (EQ-5D-3L)

Quality of life was measured twice, before and after the clinical trial, using five items, and was assessed by self-report. Table 4 presents these results. There were no significant differences within or between the groups regarding mobility, self-care, and pain/discomfort. However, in the intervention group, a statistically significant change was observed in the usual activities category between baseline and six months (*p* = 0.046), whereas no significant change was observed in the control group. Anxiety and depression showed a significant within-group change in the intervention group (*p* = 0.008) and a significant between-group difference at six months (*p* = 0.044). The quality-weighted total score did not change significantly in either the intervention or control group, and no statistically significant differences were observed within or between the groups.

## 4. Discussion

In this study, an intervention using mobile phone text messages for patients with PD over a six-month period improved several clinical parameters (HbA1c, BMI, waist circumference, LDL-C, total cholesterol, and FBG) and eating habits. Most of the other clinical values also showed favorable trends, although these changes were not statistically significant. This demonstrates that text message interventions for patients with PD can play an auxiliary role in diabetes prevention and the formation of self-management behaviors. Increased knowledge of diabetes and risk perception are important determinants of health behaviors [26], and positive changes in risk perception were observed in patients exposed to mobile phone text message interventions [27]. Telemedicine, ranging from simple text messages to complex web-based interfaces, can promote self-management, a potential goal of remote healthcare, especially in diabetes management [17], and has been associated with significant reductions in HbA1c [28].

Prevention of diabetes through lifestyle improvement remains an important priority [29]. According to Waller et al., no significant difference was observed between the intervention and control groups in terms of the effect of the mobile phone message intervention on self-management behaviors and HbA1c value improvement over six months in Australian patients with type 2 diabetes [30]. This was because the participants used medication for diabetes, which limited the potential for further HbA1c reduction through a mobile phone text messaging intervention. However, in this study, several clinical parameters (HbA1c, BMI, waist circumference, LDL-C, total cholesterol, FBG) and dietary behaviors (milk consumption and instant food consumption) showed significant changes in the intervention group between baseline and six months, which may be attributed to the fact that the study targeted patients with PD. Waller et al. also suggested that mobile phone text messaging interventions are highly likely to lead to beneficial results for patients with PD, primarily before administering drug therapy [30].

Additionally, there was a statistically significant difference in LDL-C and instant food consumption between the intervention and control groups (*p* = 0.016). This suggests that lifestyle and health interventions through text messages for patients with PD can be effective prior to the onset of pathological symptoms associated with type 2 diabetes. Although these values did not reach pathological thresholds, lifestyle behavior changes can help patients with PD develop healthy habits. In contrast, triglyceride levels showed an increasing tendency in the intervention group, although this change was not statistically significant. This finding may be related to the characteristics of the study population, which consisted of older adults engaged in agricultural activities. Agricultural work is often associated with seasonal variations in physical activity and irregular meal patterns, both of which may influence triglyceride levels [31,32]. In addition, triglycerides are also known to exhibit greater biological variability compared with LDL-C, particularly in older adults [33]. Notably, most of the statistically significant findings were more pronounced among female participants. Women generally demonstrate greater receptivity to health-related information and higher adherence to recommended lifestyle modifications than men [34]. In particular, the increase in milk consumption may reflect the adoption of a specific and easily implementable dietary alternative [35]. Such changes may also reflect a substitution effect, in which snack composition or vegetable consumption was replaced by other food items. In the control group, sex-specific differences were also observed. Late-night eating increased significantly among men, whereas instant food consumption and the frequency of eating out increased among women. These differences may reflect gender-related behavioral patterns in older adults. Men may be more likely to maintain established dietary routines, including late-night eating habits, whereas women, who often assume primary responsibility for meal preparation, may be more susceptible to convenience-oriented dietary shifts in the absence of structured intervention messages [34,36,37].

A previous study by Chow et al., which provided a text message intervention to patients with coronary artery disease for six months, reported an improvement in LDL-C levels [38], and Dobson et al. demonstrated that a text message-based self-management support program significantly reduced HbA1c levels in adults with type 2 diabetes [39]. These results are consistent with the findings of the present study. Text messaging interventions can be provided at a low cost, and as most people across all income groups own a mobile phone, they can have a significant impact on the prevention and improvement of various diseases. Consuming one small serving of sweets per week may not affect weight management or HbA1c control, but substituting vegetables, fruit, and whole grains for those foods may be beneficial [40,41].

In this study, the intervention group showed statistically significant improvements in HbA1c, BMI, waist circumference, LDL-C, total cholesterol, FBG, and milk consumption. These findings support the contribution of the intervention to the formation of healthy lifestyle habits. However, HbA1c, waist circumference, and FBG improved in both the intervention and control groups, which is considered to reflect the Hawthorne effect, as the control group was also participating in the clinical trial. The Hawthorne effect refers to the psychological tendency of participants to choose healthier behaviors than usual when they realize they are being observed; participants in the control group may also have reviewed and modified their eating habits during the evaluation process [42,43]. Additionally, the control group showed a statistically significant improvement in fruit consumption. This observation suggests that participants exposed to the experimental setting may have consciously engaged in health behaviors perceived as desirable by the researchers [44]. However, the frequency of late-night eating increased significantly in the control group, and instant food consumption showed a statistically significant difference compared with the intervention group at six months. Therefore, the dietary improvements and reductions in unhealthy habits observed in the intervention group support the effectiveness of the text message-based intervention.

Simon et al. reported that, in patients with type 2 diabetes, quality of life did not change in the control group, whereas it worsened in the self-monitoring group due to increased usual activity and anxiety/depression [45]. In contrast, in the present study, quality of life assessment showed that only the intervention group experienced an improvement in anxiety and depression, which differed significantly from the control group. Self-monitored mobile health interventions may improve mental health management in adults aged 65 years and older and reduce depressive symptoms [46]. Additionally, unhealthy diets and reduced physical activity are associated with depressive symptoms, and the formation of healthy habits positively affects the reduction in depressive symptoms [47]. The slight increase in usual activity problems may reflect greater awareness of physical activity, leading to an increased burden or perceived difficulty due to higher activity levels [48].

A limitation of this study is that investigator blinding was not applied when delivering the intervention. Additionally, the effects of a longer intervention period could not be evaluated within the six-month study duration. The distribution of participants’ age, income, and occupational characteristics was skewed toward specific subgroups, which limited the ability to adequately account for differences related to these factors. Although improvements in HbA1c were observed, the participants were classified as having PD and therefore remained within a high-risk range for developing type 2 diabetes. Despite these improvements, continued monitoring and management are necessary. Moreover, progression to type 2 diabetes may be influenced by multiple factors, including genetic predisposition, lifestyle behaviors, and metabolic conditions, which were not comprehensively controlled in this study. In addition, dietary habits were assessed through self-report with the assistance of a coordinator; however, self-reported measures are subject to recall bias and social desirability bias, which may have affected the reliability and accuracy of the findings. Nevertheless, this study is one of the few to target middle-aged and older South Koreans with PD, rather than patients with type 2 diabetes, for diabetes prevention.

Mobile phone text messaging is an innovative intervention that can be applied immediately without additional training, does not impose a burden on participants, and functions as a minimal intervention that does not substantially alter daily life. Future studies are warranted to evaluate the long-term effects of this intervention using individualized protocols over extended follow-up periods.

## 5. Conclusions

This study demonstrated that a six-month mobile phone text message-based intervention for individuals with PD was associated with improvements in selected clinical indicators and health-related behaviors. In the intervention group, significant reductions were observed in HbA1c, waist circumference, BMI, LDL-C, total cholesterol, and fasting blood glucose (FBG), along with favorable changes in dietary habits. In addition, improvements in anxiety and depression were observed in the intervention group, suggesting potential benefits for mental well-being. These results support the feasibility of text message-based interventions for promoting self-management and lifestyle modification in individuals with prediabetes.

## Figures and Tables

**Table 1:** Examples of text messages by category.

Category (N)	Examples of Text Messages
Become aware: 7 (self-reflective thoughts 4, medical knowledge 2, lifestyle 1)	- To prevent diabetes, the waist circumference should be less than 80 cm (32 inches) for women and less than 90 cm (36 inches) for men. - For a healthy life, learn a lot about diabetes. Maintain a healthy diet and regular walking activity!
Choose alternative: 9 (nutrition)	- If you are hungry between meals, eat fruit instead of snacks. Apples, bananas, or oranges are good. - Eat protein-rich foods! Low-fat milk, soy, eggs, nuts, chicken, and fish are good for your health.
Commit to change: 8 (physical activity 5, nutrition 1, medical knowledge 1, lifestyle 1)	- Set up daily physical activity as long as you are able to walk. Try to walk more than you did last time! The added step count is good for your health! - Blood glucose control can be managed with a healthy diet, including vegetables and fish and physical activity.
Control risk: 12 (medical knowledge 8, physical activity 2, nutrition 1, lifestyle 1)	- If you have diabetes in your family history, you are at risk too. Reduce your risk by taking a small walk each week. - The World Health Organization says that 80% of diabetes cases can be prevented by a healthy diet, moderate physical activity, and smoking cessation.
Seek support: 6 (lifestyle 3, medical knowledge 2, self-reflective thoughts 1)	- If you are a smoker, especially if you have been diagnosed with diabetes, quit smoking today. Seek support from family and friends too. - Send text messages to your friends to go walking, exercise, or do yoga together! Physical activity will be more enjoyable if you are with a friend as well.
Know impact: 6 (medical knowledge)	- Many medical studies have proven that daily physical activity and a healthy diet can prevent type 2 diabetes. - High blood glucose caused by diabetes causes problems with your eyes, kidneys, heart, feet, and nerves.

**Table 2:** Baseline characteristics of the intervention and control groups.

Demographics	Intervention Group (*n* = 46)	Control Group (*n* = 50)	*p*-Value
Age (years), mean ± SD	65.4 ± 5.5	66.9 ± 6.5	0.247
Age group, *n*			0.318
50–54 years	0 (0%)	2 (4%)	
55–59 years	7 (15%)	4 (8%)	
60–69 years	27 (59%)	27 (54%)	
70–79 years	12 (26%)	17 (34%)	
Gender, *n*			0.079
Male	14 (30%)	24 (48%)	
Female	32 (70%)	26 (52%)	
Clinical measures, mean ± SD			
Height (cm)	158.4 ± 8.8	159.1 ± 7.2	0.682
Weight (kg)	66.0 ± 10.3	64.5 ± 7.7	0.419
Diastolic blood pressure (mmHg)	88.7 ± 9.3	89.2 ± 8.7	0.432
Systolic blood pressure (mmHg)	140.7 ± 17.0	143.1 ± 11.8	0.784
Educational level, *n*			0.724
No formal education	0 (0%)	0 (0%)	
Elementary school	11 (24%)	7 (14%)	
Middle school	8 (17%)	10 (20%)	
High school	17 (37%)	18 (36%)	
College or higher	9 (20%)	14 (28%)	
Unknown	1 (2%)	1 (2%)	
Average monthly income, *n*			0.191
<1 million KRW (≈USD 750)	7 (15%)	4 (8%)	
1–2.99 million KRW (≈USD 750–2250)	22 (48%)	28 (56%)	
3–4.99 million KRW (≈USD 2250–3750)	13 (28%)	9 (18%)	
≥5 million KRW (≈USD 3750)	3 (7%)	9 (18%)	
Unknown	1 (2%)	0 (0%)	

Variables are presented as mean ± standard deviation or number (%), as appropriate. SD, standard deviation. *p*-values were calculated using the independent *t*-test for continuous variables and the chi-square test for categorical variables.

**Table 3:** Intervention effects on the primary and secondary visits.

		Intervention Group (*n* = 46)			Control Group (*n* = 50)			** *p*-Value
		Total	Male	Female	Total	Male	Female	
Clinical Measurements								
HbA1c (%)	Baseline	6.1 ± 0.5	5.9 ± 0.3	6.2 ± 0.5	6.1 ± 0.2	6.0 ± 0.2	6.1 ± 0.2	0.864
	Six months	6.0 ± 0.3	5.8 ± 0.2	6.1 ± 0.4	6.0 ± 0.3	6.0 ± 0.3	6.0 ± 0.2	0.686
	Change	−0.1 ± 0.4	−0.1 ± 0.1	−0.1 ± 0.4	−0.1 ± 0.2	−0.1 ± 0.2	−0.1 ± 0.2	
	* *p*-value	0.043	0.069	0.093	0.023	0.124	0.107	
Body mass index (kg/m^2^)	Baseline	26.2 ± 3.0	25.6 ± 2.8	26.5 ± 3.0	25.5 ± 2.7	25.3 ± 2.4	25.7 ± 2.9	0.199
	Six months	25.9 ± 3.0	25.2 ± 2.6	26.3 ± 3.1	25.7 ± 2.9	25.5 ± 2.8	25.9 ± 3.1	0.684
	Change	−0.3 ± 0.8	−0.4 ± 0.9	−0.2 ± 0.7	0.2 ± 1.0	0.2 ± 1.1	0.2 ± 0.8	
	* *p*-value	0.019	0.138	0.081	0.121	0.283	0.270	
Waist circumference (cm)	Baseline	91.9 ± 7.1	94.8 ± 8.3	90.6 ± 6.2	92.2 ± 5.9	93.7 ± 5.8	90.9 ± 5.7	0.792
	Six months	87.7 ± 8.2	90.1 ± 8.5	86.7 ± 8.0	89.3 ± 8.3	92.3 ± 7.5	86.6 ± 8.2	0.342
	Change	−4.1 ± 4.9	−4.7 ± 3.2	−3.9 ± 5.4	−2.9 ± 4.8	−1.4 ± 3.6	−4.2 ± 5.5	
	* *p*-value	<0.001	<0.001	<0.001	<0.001	0.065	<0.001	
High-density lipoprotein cholesterol (mg/dL)	Baseline	51.0 ± 9.9	47.2 ± 9.0	52.6 ± 10.0	48.1 ± 10.0	45.2 ± 9.3	50.8 ± 9.9	0.163
	Six months	49.4 ± 10.8	45.0 ± 10.2	51.3 ± 10.6	48.5 ± 9.7	47.5 ± 10.4	49.5 ± 9.2	0.677
	Change	−1.6 ± 9.6	−2.2 ± 6.2	−1.3 ± 10.8	0.4 ± 7.0	2.3 ± 8.2	−1.3 ± 5.2	
	* *p*-value	0.274	0.201	0.508	0.671	0.174	0.200	
Low-density lipoprotein cholesterol (mg/dL)	Baseline	115.5 ± 32.2	114.5 ± 28.7	115.9 ± 34.1	121.8 ± 35.0	127.3 ± 32.7	116.7 ± 36.9	0.362
	Six months	102.5 ± 30.9	105.1 ± 32.2	101.3 ± 30.8	119.1 ± 35.3	126.5 ± 37.8	112.3 ± 32.0	0.016
	Change	−13.0 ± 31.8	−9.4 ± 24.2	−14.6 ± 34.8	−2.7 ± 31.6	−0.8 ± 28.5	−4.4 ± 34.8	
	* *p*-value	0.007	0.171	0.023	0.554	0.898	0.522	
Triglycerides (mg/dL)	Baseline	137.8 ± 54.0	152.0 ± 63.2	131.6 ± 49.3	168.4 ± 89.8	159.3 ± 57.8	176.8 ± 112.1	0.048
	Six months	149.0 ± 65.0	182.2 ± 86.7	134.4 ± 47.5	157.9 ± 83.0	140.8 ± 77.8	173.7 ± 85.9	0.562
	Change	11.2 ± 48.6	30.2 ± 70.5	2.8 ± 33.4	−10.5 ± 93.8	−18.5 ± 72.7	−3.1 ± 110.7	
	* *p*-value	0.126	0.132	0.633	0.432	0.224	0.888	
Total cholesterol (mg/dL)	Baseline	190.0 ± 37.1	187.7 ± 35.3	191.0 ± 38.3	196.8 ± 36.1	196.0 ± 34.5	197.6 ± 38.1	0.365
	Six months	174.1 ± 33.4	178.1 ± 37.6	172.4 ± 31.9	191.7 ± 35.9	191.6 ± 39.4	191.9 ± 33.2	0.014
	Change	−15.9 ± 33.2	−9.6 ± 25.8	−18.7 ± 36.0	−5.1 ± 30.2	−4.4 ± 26.4	−5.7 ± 33.9	
	* *p*-value	0.002	0.184	0.006	0.240	0.425	0.396	
Fasting blood glucose (mg/dL)	Baseline	102.5 ± 10.5	107.1 ± 8.9	100.5 ± 10.6	102.4 ± 9.7	105.2 ± 11.3	99.9 ± 7.4	0.960
	Six months	98.9 ± 11.8	104.4 ± 14.4	96.5 ± 9.7	98.7 ± 10.0	101.8 ± 10.9	95.9 ± 8.4	0.931
	Change	−3.6 ± 11.5	−2.7 ± 15.8	−4.0 ± 9.4	−3.7 ± 8.9	−3.4 ± 10.6	−4.0 ± 7.1	
	* *p*-value	0.038	0.530	0.021	0.004	0.127	0.008	
Health behaviors								
Grain consumption (times/day)	Baseline	2.5 ± 0.8	2.2 ± 0.9	2.6 ± 0.7	2.4 ± 0.9	2.4 ± 0.9	2.4 ± 0.9	0.529
	Six months	2.2 ± 1.1	2.1 ± 1.2	2.3 ± 1.0	2.2 ± 1.1	2.4 ± 0.9	2.0 ± 1.2	0.822
	Change	−0.3 ± 1.1	−0.1 ± 1.0	−0.3 ± 1.1	−0.2 ± 1.4	0.0 ± 1.3	−0.4 ± 1.4	
	* *p*-value	0.08	0.640	0.089	0.252	0.963	0.154	
Fish consumption (times/day)	Baseline	1.0 ± 0.8	1.0 ± 0.6	1.0 ± 0.8	1.1 ± 0.9	1.2 ± 1.0	0.9 ± 0.7	0.632
	Six months	1.1 ± 0.9	1.1 ± 0.9	1.1 ± 0.9	1.2 ± 1.0	1.2 ± 1.0	1.2 ± 1.0	0.684
	Change	0.1 ± 1.0	0.2 ± 0.7	0.1 ± 1.2	0.1 ± 0.9	0.0 ± 0.8	0.3 ± 0.9	
	* *p*-value	0.341	0.427	0.489	0.253	0.810	0.083	
Fruit consumption (times/day)	Baseline	1.1 ± 0.7	0.9 ± 0.6	1.2 ± 0.7	0.9 ± 0.5	0.9 ± 0.4	0.9 ± 0.5	0.055
	Six months	1.2 ± 0.8	1.1 ± 0.9	1.2 ± 0.8	1.1 ± 0.7	1.1 ± 0.7	1.1 ± 0.7	0.785
	Change	0.1 ± 0.8	0.2 ± 0.8	0.0 ± 0.8	0.2 ± 0.7	0.3 ± 0.7	0.2 ± 0.6	
	* *p*-value	0.609	0.483	0.881	0.010	0.069	0.079	
Vegetable consumption (times/day)	Baseline	2.0 ± 1.0	1.5 ± 0.9	2.2 ± 1.0	1.9 ± 1.1	2.0 ± 1.1	1.9 ± 1.1	0.907
	Six months	1.6 ± 1.0	1.5 ± 1.0	1.6 ± 1.0	1.8 ± 1.0	1.7 ± 1.0	1.9 ± 1.0	0.236
	Change	−0.4 ± 1.3	0.0 ± 1.3	−0.6 ± 1.3	−0.1 ± 1.2	−0.3 ± 1.3	0 ± 1.1	
	* *p*-value	0.050	1	0.022	0.508	0.356	0.961	
Milk consumption (times/day)	Baseline	0.6 ± 0.4	0.6 ± 0.4	0.6 ± 0.4	0.8 ± 0.7	0.8 ± 0.6	0.7 ± 0.7	0.112
	Six months	0.8 ± 0.6	0.8 ± 0.3	0.8 ± 0.7	0.9 ± 0.7	0.9 ± 0.7	0.8 ± 0.7	0.640
	Change	0.2 ± 0.5	0.2 ± 0.4	0.2 ± 0.6	0.1 ± 0.7	0.1 ± 0.8	0.1 ± 0.5	
	* *p*-value	0.004	0.088	0.020	0.233	0.574	0.179	
Fatty food consumption (times/week)	Baseline	1.4 ± 1.2	1.5 ± 1.3	1.3 ± 1.1	1.2 ± 1.0	1.5 ± 1.2	1.4 ± 0.7	0.803
	Six months	1.4 ± 1.0	1.6 ± 1.0	1.3 ± 1.1	1.3 ± 0.8	1.3 ± 0.9	1.4 ± 0.7	0.688
	Change	0.0 ± 1.3	0.1 ± 1.3	0.0 ± 1.3	−0.1 ± 0.9	−0.2 ± 1.1	0.0 ± 0.7	
	* *p*-value	0.954	0.693	0.837	0.347	0.322	0.840	
Instant food consumption (times/week)	Baseline	0.7 ± 0.8	0.9 ± 0.9	0.7 ± 0.7	1.0 ± 0.8	1.2 ± 0.8	0.8 ± 0.8	0.158
	Six months	0.7 ± 0.8	1.0 ± 0.9	0.6 ± 0.7	1.1 ± 1.1	1.1 ± 1.2	1.2 ± 1.0	0.035
	Change	0.0 ± 0.9	0.1 ± 1.1	−0.1 ± 0.7	0.2 ± 1.1	−0.1 ± 1.2	0.4 ± 1.0	
	* *p*-value	0.800	0.637	0.414	0.333	0.648	0.047	
Junk food consumption (times/week)	Baseline	0.4 ± 0.6	0.5 ± 0.6	0.4 ± 0.6	0.4 ± 0.5	0.4 ± 0.6	0.5 ± 0.5	0.879
	Six months	0.3 ± 0.5	0.3 ± 0.4	0.3 ± 0.5	0.5 ± 0.7	0.5 ± 0.6	0.5 ± 0.7	0.081
	Change	−0.1 ± 0.5	−0.2 ± 0.7	−0.1 ± 0.5	0.1 ± 0.6	0.1 ± 0.5	0.1 ± 0.6	
	* *p*-value	0.198	0.309	0.445	0.268	0.408	0.468	
Late-night eating (times/week)	Baseline	0.4 ± 1.0	0.4 ± 1.1	0.4 ± 0.9	0.3 ± 0.6	0.3 ± 0.6	0.3 ± 0.7	0.460
	Six months	0.5 ± 0.9	0.5 ± 0.7	0.6 ± 1.0	0.8 ± 1.5	1.0 ± 1.4	0.6 ± 1.6	0.287
	Change	0.1 ± 1.3	0.1 ± 1.3	0.1 ± 1.3	0.5 ± 1.5	0.8 ± 1.2	0.3 ± 1.7	
	* *p*-value	0.478	0.758	0.526	0.014	0.006	0.343	
Take away food(times/week)	Baseline	1.6 ± 1.1	2.0 ± 1.0	1.4 ± 1.1	1.6 ± 1.2	1.7 ± 1.6	1.4 ± 0.8	0.990
	Six months	1.6 ± 1.1	2.1 ± 1.2	1.3 ± 1.0	1.8 ± 1.3	1.5 ± 1.0	2.1 ± 1.5	0.343
	Change	0.0 ± 1.2	0.1 ± 1.1	0.0 ± 1.3	0.2 ± 1.4	−0.3 ± 1.4	0.7 ± 1.4	
	* *p*-value	1	0.854	0.919	0.238	0.374	0.015	
Overeating (times/week)	Baseline	1.2 ± 1.6	1.1 ± 1.3	1.3 ± 1.8	1.4 ± 1.6	1.5 ± 2.0	1.2 ± 1.1	0.669
	Six months	1.5 ± 1.5	1.3 ± 1.5	1.6 ± 1.5	1.5 ± 1.7	1.4 ± 1.6	1.7 ± 1.8	0.851
	Change	0.2 ± 2.2	0.1 ± 1.8	0.3 ± 2.3	0.2 ± 1.7	−0.1 ± 1.8	0.4 ± 1.6	
	* *p*-value	0.457	0.775	0.497	0.507	0.733	0.190	

Variables are presented as mean ± standard deviation. Dietary intake variables were calculated as times per day or week. * *p*-values represent within-group comparisons between baseline and six months; ** *p*-values represent comparisons between the intervention and control groups.

**Table 4:** Changes in quality of life in the intervention and control groups.

Demographics	Intervention Group (*n* = 46)				Control Group (*n* = 50)				** *p*-Value
	No Problem	Some Problem	Unable	* *p*-Value	No Problem	Some Problem	Unable	*p*-Value	
Mobility				0.705				0.705	
Baseline	35	11	0		40	10	0		0.643
Six months	34	12	0		39	11	0		0.639
Self-Care				0.317				1.000	
Baseline	46	0	0		47	5	1		0.241
Six months	45	1	0		47	2	1		0.545
Usual activity				0.046				0.480	
Baseline	45	1	0		44	6	0		0.064
Six months	41	5	0		42	8	0		0.463
Pain/Discomfort				0.405				0.782	
Baseline	26	20	0		24	24	2		0.320
Six months	23	23	0		26	21	3		0.211
Anxiety/Depression				0.008				0.705	
Baseline	35	11	0		39	11	0		0.824
Six months	42	4	0		38	12	0		0.044
Quality-weighted total score				0.847				0.642	
Baseline		0.78 ± 0.22				0.68 ± 0.41			0.137
Six months		0.78 ± 0.25				0.65 ± 0.53			0.148

Variables are presented as numbers unless otherwise indicated. The quality-weighted total score (EQ-5D-3L index) is presented as mean ± standard deviation. * *p*-values represent within-group comparisons between baseline and six months; ** *p*-values represent comparisons between the intervention and control groups.

## Data Availability

The data presented in this study are available from the corresponding author upon reasonable request. The data are not publicly available due to privacy restrictions.
